# Wide-Field Megahertz OCT Imaging of Patients with Diabetic Retinopathy

**DOI:** 10.1155/2015/305084

**Published:** 2015-07-27

**Authors:** Lukas Reznicek, Jan P. Kolb, Thomas Klein, Kathrin J. Mohler, Wolfgang Wieser, Robert Huber, Marcus Kernt, Josef Märtz, Aljoscha S. Neubauer

**Affiliations:** ^1^Department of Ophthalmology, Technical University of Munich, Ismaninger Strasse 22, 81675 Munich, Germany; ^2^Institute for Biomolecular Optics, Ludwig Maximilians University, Oettingenstrasse 67, 80538 Munich, Germany; ^3^Institute of Biomedical Optics, University of Lübeck, Peter-Monnik-Weg 4, 23562 Lübeck, Germany; ^4^Department of Ophthalmology, Ludwig Maximilians University, Mathildenstrasse 8, 80336 Munich, Germany

## Abstract

*Purpose*. To evaluate the feasibility of wide-field Megahertz (MHz) OCT imaging in patients with diabetic retinopathy. *Methods*. A consecutive series of 15 eyes of 15 patients with diagnosed diabetic retinopathy were included. All patients underwent Megahertz OCT imaging, a close clinical examination, slit lamp biomicroscopy, and funduscopic evaluation. To acquire densely sampled, wide-field volumetric datasets, an ophthalmic 1050 nm OCT prototype system based on a Fourier-domain mode-locked (FDML) laser source with 1.68 MHz A-scan rate was employed. *Results*. We were able to obtain OCT volume scans from all included 15 patients. Acquisition time was 1.8 seconds. Obtained volume datasets consisted of 2088 × 1044 A-scans of 60° of view. Thus, reconstructed en face images had a resolution of 34.8 pixels per degree in *x*-axis and 17.4 pixels per degree. Due to the densely sampled OCT volume dataset, postprocessed customized cross-sectional B-frames through pathologic changes such as an individual microaneurysm or a retinal neovascularization could be imaged. *Conclusions*. Wide-field Megahertz OCT is feasible to successfully image patients with diabetic retinopathy at high scanning rates and a wide angle of view, providing information in all three axes. The Megahertz OCT is a useful tool to screen diabetic patients for diabetic retinopathy.

## 1. Introduction

Diabetic retinopathy is a leading cause for blindness in industrialized countries [1]. The prevalence of diabetes and diabetic retinopathy is increasing worldwide including first world countries [2]. Thus, early detection of diabetic changes of the posterior ocular fundus for an individualized and optimized treatment is of high importance.

Multiple noninvasive diagnostic imaging tools for screening as well as detection and monitoring of the diabetic retinopathy are available, for example, fundus cameras [3, 4] or scanning laser ophthalmoscopy [5] for en face images of the posterior fundus or OCT scans for cross-sectional images of the central retina [6–8].

In general, retinal diseases such as diabetic retinopathy can manifest in the central as well as peripheral retina and can initially develop subtle pathologic alterations. The NHS Diabetic Eye Screening Program in the United Kingdom as well as the conclusions of Early Treatment Diabetic Retinopathy Study, for example, demands a field of view of 45°  × 40° for fundus cameras to ensure high sensitivity and specificity to successfully screen for diabetic retinopathy. Therefore, the requirements for valid images to effectively depict the posterior fundus are a wide angle of view and a high resolution. Fundus cameras fulfilling those requirements are able to obtain en face images in *x*-*y*-axis with useful information depicting potential diabetic pathologies located on the surface of the retina. On the other hand, commercially available OCT devices have been able to obtain cross-sectional scans of the central retina but were limited to a narrow angle of view (30 × 20°) and slow acquisition speed, thus failing to provide enough pixels per degree in reconstructed en face display modes.

The recently introduced ultrahigh-speed swept source Fourier-domain mode-locked (FDML) OCT [9–11] is feasible at high scanning rates providing images with a wide angle of view. The obtained volume scans of the retina allowed a reconstruction of high resolution en face images as well as histology-like cross-sectional retinal scans (B-frames) depicting the fundus beyond the central macular area with an image quality comparable to current established OCT devices [9].

The purpose of this study was to evaluate the feasibility of this novel device imaging diabetic patients with diagnosed diabetic retinopathy.

## 2. Patients and Methods

We retrospectively selected 15 eyes of 15 consecutive patients with diagnosed diabetic retinopathy (5 eyes with proliferative diabetic retinopathy versus 10 eyes with nonproliferative diabetic retinopathy) who were imaged between October 2013 and April 2014 in the MHz OCT study at the LMU Eye Hospital Munich (German Clinical Trial Register ID DRKS00005173). Three were females and 12 were males. Mean age was 57.4 ± 11.1 years (range 41–81 years). Ethics committee approval was obtained in June 2013. Written informed consent was obtained from all patients prior to OCT imaging. The research adhered to the tenets of the Declaration of Helsinki.

All patients underwent Megahertz OCT imaging, a close clinical examination including measurements of refraction, best corrected VA, slit lamp biomicroscopy, and funduscopic evaluation. Patients were not dilated specifically for the imaging procedures. Only one eye per subject was selected to avoid any intraindividual correlations.

The ophthalmic OCT prototype system is based on a Fourier-domain mode-locked (FDML) laser source, which makes the acquisition of densely sampled, wide-field volumetric datasets possible. Details of this custom FDML MHz OCT system can be found in [9, 12, 13].

In summary, the system employs a rapidly wavelength-tunable FDML laser with a center wavelength of 1050 nm for higher tissue penetration in contrast to spectral-domain OCT. It can obtain an ultrahigh number of 1.68 Mio depth scans (A-scans) per second. The sweep bandwidth of approximately 70 nm makes an axial resolution of 10 *μ*m in tissue possible. 1.6 mW sample arm power is incident on the eye, which is below the limits declared in the American National Standard Institute (ANSI) standards for safe ocular exposure (ANSI, “Safe Use of Lasers & Safe Use of Optical Fiber Communications,” American National Standard Institute, Z136 Committee). At this power, the device achieves a sensitivity of 91 dB.

## 3. Results

**Figure 1:**
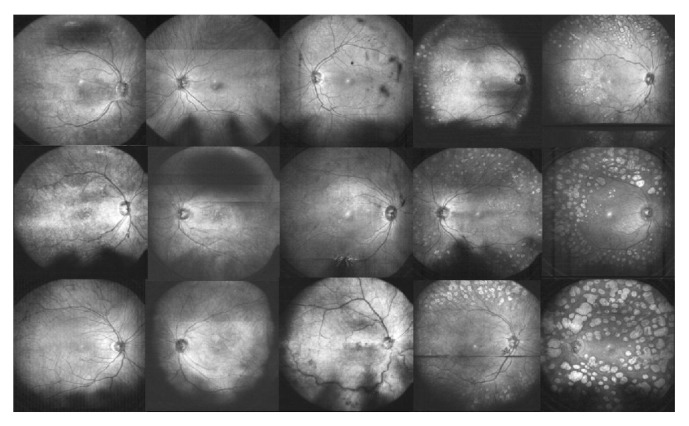
Reconstructed en face wide-field fundus images of all 15 patients; artifacts are shown in detail in Figure 2.

We were able to obtain OCT volume scans from all included 15 patients. Acquisition time was 1.8 seconds. Two-dimensional en face images were reconstructed by taking the average of each A-scan of the three-dimensional OCT imaging data to provide a first overview. Then, arbitrary cross-sectional views can be obtained at every location for a closer examination as shown later on. Wide-field en face images from all patients can be seen in Figure 1.

**Figure 2:**
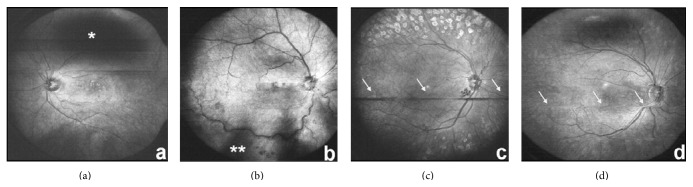
Artifacts of reconstructed en face wide-field fundus images with pupillary shadowing in (a) (*∗*) and ciliary shadowing in (b) (*∗∗*). Eye blinks result in horizontal black stripes seen in (c) and saccades in misalignments (see (d)) in the reconstructed en face images.

Artifacts and possible quality modulations were pupillary or ciliary shadowing as known from other wide-field devices; motion artifacts such as eye blinks result in horizontal black stripes while saccades result in a “misalignment phenomenon” of the reconstructed en face images; see Figure 2. Ciliary shadowing is the only observed artifact that can be solved with pupil dilation.

**Figure 3:**
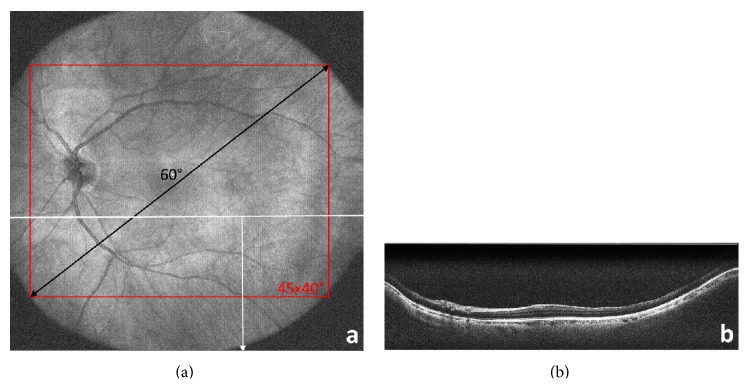
OCT images cover a field of view with a diameter of 60° which corresponds to 45°  × 40° as shown in the en face reconstruction in (a). Cross-sectional views (B-frames) can be obtained at every location as seen in (b).

Angle of view of the OCT imaging data was 60° in diameter including the macular area, optic disc, and midperipheral retina significantly beyond the major vessel arcades; see Figure 3. For example, the NHS Diabetic Eye Screening Program in the United Kingdom demands a field of view of 45°  × 40° for fundus cameras, which is also covered by the three-dimensional data of the Megahertz OCT.

**Figure 4:**
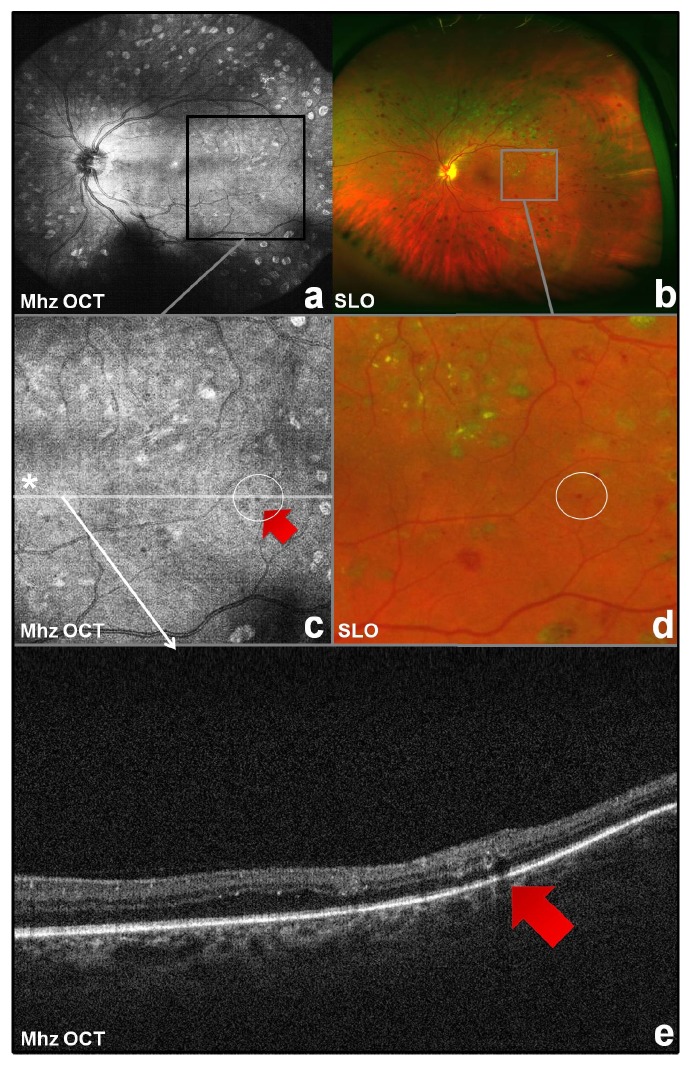
Reconstructed MHz OCT en face wide-field fundus image of left eye (a) with detail (c) versus wide-field scanning laser ophthalmoscopy (SLO) image (b) with detail (d). Cross-sectional MHz OCT scan from the same dataset (a, c) through a microaneurysm (red arrow) providing information in *z*-axis (e).

Obtained volume datasets consisted of 2088 × 1044 A-scans with 60° field of view. Thus, reconstructed en face images had a resolution of 34.8 pixels per degree in *x*-axis and 17.4 pixels per degree in *y*-axis revealing fine details such as retinal microaneurysms (see Figures 4(a) and 4(c)).  Figure 4(e) shows a cross-sectional scan of the imaged retina through a microaneurysm providing information in *z*-axis. Figures 4(b) and 4(d) display images of the same eye obtained with a wide-field scanning laser ophthalmoscope (Optos plc, Dunfermline, UK) serving as a reference.

**Figure 5:**
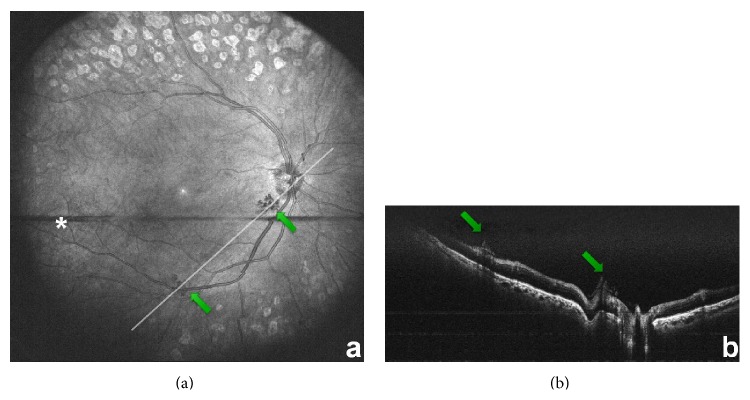
Reconstructed MHz OCT en face wide-field fundus image of right eye (a) with neovascularisations (green arrows); star shows blinking as horizontal black stripe; line represents cross-sectional scan of (b). (b) shows B-frame obtained in postprocessing through the neovascularisations seen in (a), with green arrows pointing at neovascularisations. The two horizontal lines are an artifact from the image processing.

Additionally, customized postprocessed B-frames depict retinal or choroidal regions of interest in *x*-*y*- and *z*-axis; see Figure 5.

Pathologic changes such as an individual microaneurysm or a retinal neovascularisation could be imaged in arbitrary customized cross-sections extracted from to the densely sampled OCT volume dataset.

## 4. Discussion

In this study, for the first time, we were able to obtain images of the posterior fundus of patients with diabetic retinopathy applying the recently [9] novel wide-field Megahertz OCT device. Volume scans from all imaged patients allowed for reconstructed high resolution en face images of the central and midperipheral fundus as well as cross-sectional scans of the retinal area of interest. A wide field of view is important for diagnostic tools used for retinal diseases to depict alterations located beyond the central posterior fundus, such as in the presented case series of patients with diabetic retinopathy.

Given the acquisition speed of 1.68 MHz (1.680.000 A-scans per second), the obtained volume scans are densely sampled, provide a sufficient angle of view of the central as well as midperipheral fundus, and result in high resolution images of each customized postprocessed arbitrary B-scan extracted from the volume scan.

Commercially available noninvasive wide-field fundus devices are able to successfully depict the posterior fundus of patients with diabetic retinopathy in *x*-*y*-axis [14] but cannot provide any information in *z*-axis. This means that devices imaging en face posterior fundus are not able to depict potential pathologies that do not show at the surface of the retina. The obtained MHz OCT volume scans of the retina on the other side allow the reconstruction of en face images which have been shown to exhibit comparable image quality to commercially available wide-field fundus devices [9] and have the additional significant advantage to provide information of the depicted area with cross-sectional scans also in *z*-axis. Cross-sectional images of patients with diabetic retinopathy obtained with the MHz OCT are therefore able to depict pathomorphologic alterations such as microaneurysms, neovascularizations (Figures 4 and 5), or intraretinal fluid and can identify their extension within middle or outer retinal layers. This additional information is missing in fundus photography due to the information provided only in *x*-*y*-axis.

The challenges with this new technology are the above mentioned ciliary and papillary shadowing which is known from other wide-field devices and motion artifacts (eye blinks, saccades) due to an acquisition time of 1.8 seconds, which in future could be reduced with faster beam scanners. Given the known inverse relationship between acquisition speed and obtained sensitivity, the achieved signal intensity of the acquired images can be significantly improved by averaging processes feasible at scanning rates beyond 1.000.000 A-scans per second and by applying higher optical power.

Limitations of our pilot study were the small number of 15 patients which was also limited by our ethics committee because of the pilot character of our study design. Further studies with large number of patients will have to confirm the feasibility. A further development of the presented MHz OCT device on the other hand will facilitate a higher competition with other diagnostic tools such as fundus cameras for screening purposes which require diagnostic tools that are easy to handle and inexpensive and create data volumes that can be transferred easily within an established network.

In summary, we were able to demonstrate the feasibility of the wide-field Megahertz OCT to successfully image patients with diabetic retinopathy at high scanning rates and a wide angle of view, providing information in all three axes. The Megahertz OCT is a useful tool to screen diabetic patients for diabetic retinopathy.
